# Treatment persistence in patients with schizophrenia treated with lurasidone in Italian clinical practice

**DOI:** 10.1186/s12991-022-00425-y

**Published:** 2022-12-16

**Authors:** Andrea Fagiolini, Miriam Olivola, Lisa Lavatelli, Antonello Bellomo, Caterina Lobaccaro, Nathalie Falsetto, Marco Micillo, Alessandro Cuomo

**Affiliations:** 1 Università Di Siena, V.Le Mario Bracci, 16, 53100 Siena (SI), Italy; 2 Department of Brain and Behavioral Sciences, University of Pavia and Servizio Psichiatrico Di Diagnosi E Cura ASST Pavia, IRCCS Policlinico San Matteo, Viale Repubblica 34, 27100 Pavia, Italy; 3 Psichiatria 1 ASST Grande Ospedale Metropolitano Niguarda, Piazza Dell’Ospedale Maggiore, 3, 20162 Milan, MI Italy; 4 University of Foggia, Viale Luigi Pinto, 1, 71122 Foggia, MI Italy; 5 Centro Salute Mentale Area 2 DSM ASL, Bari, Italy; 6 Angelini Pharma S.P.A, Viale Amelia70, 00181 Rome, Italy; 7 University of Siena, V.Le Mario Bracci, 16, 53100 Siena, SI Italy

**Keywords:** Schizophrenia, Lurasidone, Treatment persistence

## Abstract

**Background and rationale:**

Treatment persistence combines clinician and patient judgment of efficacy, tolerability and safety into a comprehensive measure of effectiveness and is defined as the act of continuing a treatment over time. Studies have reported poor treatment persistence to antipsychotic medications in patients with schizophrenia. This study evaluated treatment persistence to lurasidone (LUR) in patients with schizophrenia in a real-world Italian setting.

**Methods:**

This was a retrospective observational study of patients with schizophrenia who started treatment with LUR ≥ 6 months before inclusion. Following informed consent, data were collected starting from the index date (start of LUR treatment) at all visits occurring as per clinical practice. The primary endpoint was treatment persistence during the first 6 months, defined as the time between index date and all-cause discontinuation. Patients treated with LUR > 180 days were considered persistent. As secondary endpoint, treatment persistence was evaluated for a period of  ≥ 18 months.

**Results:**

Forty-five patients were enrolled and 41 (91.11%) completed the study. Forty-one patients (91.11%) were included in the eligible population as they initiated LUR treatment  ≥ 6 months before data collection.

Patients were 43.0 ± 15.89 years old and 61% were female. Twenty-two patients (53.66%) started LUR treatment in a hospital setting and 19 (46.34%) in an outpatient setting. Based on Clinical Global Impression—Severity scale (CGI-S) at LUR initiation, 12 patients (29.27%) were severely ill, 17.07% markedly ill, 19.51% moderately ill, 2.44% mildly ill and 4.88% borderline mentally ill. Thirty-two patients (78.05%) were treatment persistent for  ≥ 180 days. Among the 19 patients observed for  ≥ 18 months, 11 (57.89%) were persistent for  ≥ 18 months. Among the 22 study patients observed for  < 18 months, 12 (54.54%) were persistent. An improvement in schizophrenia severity according to CGI-S was observed at inclusion (following LUR therapy) compared to the index date. Six patients (14.63%) experienced at least one adverse drug reaction: akathisia (7.32%), extrapyramidal disorder (4.88%), hyperprolactinemia (2.44%), restlessness (2.44%), and galactorrhea (2.44%). None were serious.

**Conclusions:**

Persistence to LUR in patients with schizophrenia was relatively high: 78% and 58% of patients were still on LUR after 6 and 18 months of treatment, respectively. This may reflect LUR’s relatively favorable balance between efficacy and tolerability, as well as favorable patient satisfaction and acceptance.

## Background

Several studies suggest that many patients with schizophrenia discontinue treatment due to lack of efficacy, side effects or non-adherence [1, 2]. Treatment persistence refers to the act of continuing a treatment over time, i.e., to the duration of time from the initiation to the discontinuation of a treatment. Treatment persistence provides an indirect measure of efficacy and tolerability, given that a medication that is continued over a long period is more likely efficacious and well tolerated than a medication that is quickly discontinued [3].

Schizophrenia is one of the most difficult psychiatric disorders to treat, largely because of the variety and complexity of the symptoms that characterize this disease, such as delusions, hallucinations, disorganized speech and behavior, negative symptoms and cognitive impairment, which affect functioning, mental state, behaviors and relationships [4, 5].

For instance, the Clinical Antipsychotic Trials of Intervention Effectiveness (CATIE) study compared the effectiveness of antipsychotic drugs in 1493 patients with schizophrenia randomly assigned to receive olanzapine (7.5–30 mg per day), perphenazine (8–32 mg per day), quetiapine (200–800 mg per day), risperidone (1.5–6.0 mg per day), or ziprasidone (40–160 mg per day) for a period of up to 18 months. The primary outcome measure was the discontinuation of treatment due to any cause and only 26% of CATIE study subjects were persistent to their antipsychotic for at least 18 months [1].

Our retrospective observational study was designed to evaluate treatment persistence with lurasidone (LUR) in patients with schizophrenia who received LUR in a real-world Italian setting and initiated this medication at least 6 months before their inclusion in the study. Although the study observation period is shorter than the overall duration of the CATIE trial (18 months), the results provided pilot data for a study with a longer observation period. Nonetheless, indications of treatment persistence at 18 months were also evaluated in this study.

## Methods

This was a multicentric, retrospective, observational study of patients with schizophrenia aged  ≥ 18 years at the time of LUR initiation who started treatment with LUR as per normal clinical practice at least 6 months before the inclusion visit.

The study was approved by the University of Siena Institutional Review Board—Tuscany Area Vasta South-East Ethical Committee, prot 20068-28/6/2021. The primary objective was to evaluate the number of patients who continued treatment with LUR for at least 6 months. Treatment persistence was defined as the time (number of days) between the index date (initiation of LUR treatment) and all-cause discontinuation of LUR therapy. Subjects treated for more than 180 days (6 months) were considered persistent.

Specifically, we recruited patients who had started lurasidone at least 6 months before the study inclusion visit and we retrospectively evaluated how many had continued lurasidone for at least 6 months. As a secondary analysis, we also looked at the persistence in patients who had started lurasidone at least 18 months before the study inclusion visit.

Data from each patient were collected after informed consent signature and included retrospective information starting from initiation of LUR treatment (index date). Data were collected retrospectively from all visits occurring as per clinical practice (usually once monthly).

If the inclusion visit took place more than 6 months after the index date, the primary objective was assessed using only data from the first 6 months after the index date. Secondary objectives were assessed using all collected data. The study was conducted in the Department of Psychiatry of the following Italian centers: Siena (Coordinating Center), Bari, Foggia, Milano, and Pavia.

Continuous data are summarized by mean, standard deviation, median, first and third quartiles, minimum and maximum. Categorical data are presented by absolute and relative frequencies (n and %) or contingency tables. All statistical tables, listings, figures and analyses were performed by means of SAS^®^ release 9.4 or later (SAS Institute, Inc., Cary, NC, USA). A two-sided alpha level 0.05 was considered. No alpha level adjustment was carried out for primary and secondary outcome variables. Adverse events and relevant medical history were coded using the MedDRA dictionary. Medications were coded using the WHO Drug ATC Classification System.

The primary endpoint is the persistence of LUR treatment during the first 6 months after treatment initiation, defined as the time between index date (initiation of treatment) and all-cause discontinuation. Patients who were treated with LUR for more than 180 days were considered persistent.

All collected data are presented using summary statistics.

## Results

### Participants

A total of 45 patients provided written informed consent to participate in this study. Three patients (6.67%) had missing status because their source data were invalid, and one patient (2.22%) was discontinued from the study because the inclusion criteria were not met. Forty-one patients (91.11%) were considered as the eligible study population.

### Demographic and baseline characteristics

Demographic data for the eligible population are summarized in Table 1. Mean age was 43.0 ± 15.89 years (min; max: 20; 73 years) and 60.98% of patients were female. About half of the study subjects were single (48.78%) whereas 31.71% were married. Twenty-two patients (53.66%) had a high school diploma, whereas only 7 (17.07%) had a university degree. With regard to employment, 15 patients (36.59%) were unemployed, 19.51% had a full-time job, 17.07% were disabled, 12.20% were retired and another 12.20% had a part-time job. Twenty-two patients (53.66%) lived with family, 26.83% with a partner, 17.07% alone and 2.44% in a residential facility. Twenty-eight patients (68.29%) had family support (Table 1).

**Table 1:** Demographic data

	Total (*N* = 41)
Age (years)	
*n*	41
Mean (SD)	43.0 (15.89)
Median	47.0
Q1; Q3	29.0; 56.0
Range	20; 73
Sex, *n* (%)	
Male	16 (39.02)
Female	25 (60.98)
Marital status, *n* (%)	
Married	13 (31.71)
Widowed	1 (2.44)
Separated	2 (4.88)
Divorced	2 (4.88)
Single	20 (48.78)
Other	3 (7.32)
Education level, *n* (%)	
Elementary school	4 (9.76)
Middle school	8 (19.51)
High school	22 (53.66)
University degree	7 (17.07)
Occupation, *n* (%)	
Full time	8 (19.51)
Part-time	5 (12.20)
Unemployed	15 (36.59)
Disabled	7 (17.07)
Retired	5 (12.20)
Other	1 (2.44)
Living situation, *n* (%)	
Alone	7 (17.07)
With partner	11 (26.83)
With family	22 (53.66)
Residential facility	1 (2.44)
Family support, *n* (%)	
No	13 (31.71)
Yes	28 (68.29)

Percentages were computed on the eligible population

*Q1* first quartile, *Q3* third quartile, *SD* standard deviation

Mean ± SD time since schizophrenia diagnosis was 11.8 ± 12.26 years. Fifteen patients (36.59%) started LUR treatment because of the lack of efficacy of a previous drug, 12 (29.27%) because of compliance issues with a previous drug, and 6 (14.63%) because of tolerability problems. Five patients (12.20%) started treatment with LUR as first choice treatment and 3 (7.32%) because of other reasons. Twenty-two patients (53.66%) started LUR treatment in a hospital setting and the other 19 (46.34%) in an outpatient setting. Based on CGI-S measurement at LUR initiation, 12 patients (29.27%) were severely ill, 17.07% markedly ill, 19.51% moderately ill, 2.44% mildly ill and 4.88% borderline mentally ill (Table 2).

**Table 2:** Clinical and treatment characteristics

	Total (*N* = 41)
Time since schizophrenia diagnosis (years)	
*n*	41
Mean (SD)	11.8 (12.26)
Median	6.5
Q1; Q3	0.8; 20.4
Range	0; 39
Reason to initiate LUR treatment, *n* (%)	
First choice treatment	5 (12.20)
Previous drugs adherence problems	12 (29.27)
Previous drugs lack of efficacy	15 (36.59)
Previous drugs tolerability problems	6 (14.63)
Other	3 (7.32)
Health setting at LUR initiation, *n* (%)	
Hospital	22 (53.66)
Outpatient visit	19 (46.34)
CGI-S measurement at lurasidone initiation, *n* (%)	
Borderline mentally ill	2 (4.88)
Mildly ill	1 (2.44)
Moderately ill	8 (19.51)
Markedly ill	7 (17.07)
Severely ill	12 (29.27)
Missing	11 (26.83)

Index date is defined as LUR start date. Time from diagnosis is defined as: index date—date of diagnosis + 1. Percentages were computed on the eligible population

*CGI-S* Clinical Global Impression—Severity scale, *LUR* lurasidone, *Q1* first quartile, *Q3* third quartile, *SD* standard deviation

Based on CGI-S measurement at LUR initiation, 12 patients (29.27%) were severely ill, 17.07% markedly ill, 19.51% moderately ill, 2.44% mildly ill and 4.88% borderline mentally ill.

### Outcome data and main results

**Fig. 1:**
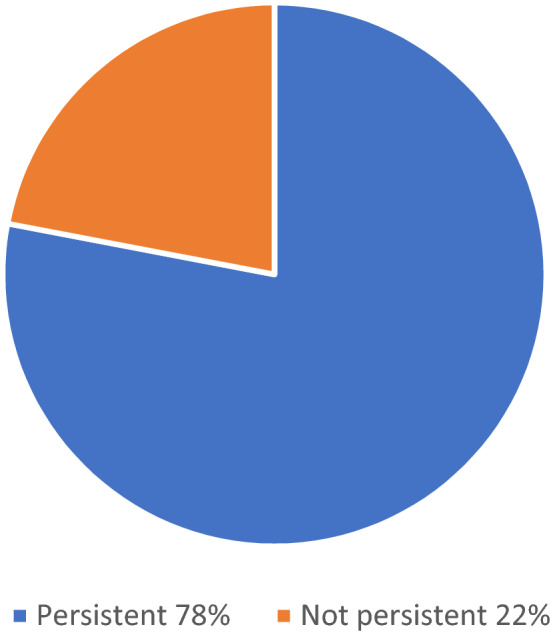
Persistence to LUR for at least 6 months

The primary endpoint was the persistence to LUR treatment during the first 6 months after LUR treatment initiation. Thirty-two patients (78%) were persistent to LUR treatment for at least 6 months (Fig. 1). The mean time to LUR discontinuation in the non-persistent patients was 59.11 ± 55.85 days (min; max: 9; 171 days).

**Fig. 2:**
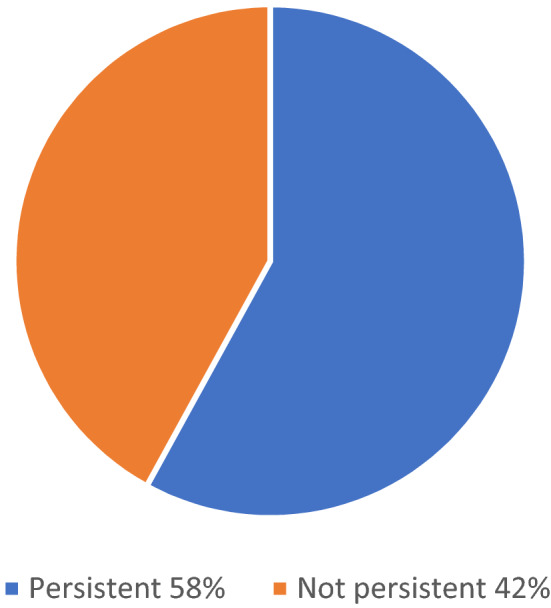
Persistence to LUR for at least 18 months

Of the 19 patients who were observed for more than 18 months after the visit when they started LUR, 11 (58%) were persistent to LUR treatment for at least 18 months (Fig. 2). The mean time to LUR discontinuation in the non-persistent patients was 267.88 ± 182.42 days (min; max: 13; 525 days).

None of the demographic and clinical variables assessed were significantly predictive of time to discontinuation of LUR treatment (*p* > 0.05). The mean number of days of LUR treatment was 461.9 ± 418.87 (min; max: 9; 1684), the mean daily dose 94.6 ± 41.63 mg (min; max: 19; 148 mg). Twenty-eight patients had a lurasidone dose increase in the observed period, whereas 11 patients had a dose reduction.

Nineteen patients (46.34%) discontinued LUR treatment: 7 patients (36.84%) because of lack of efficacy, 4 (21.05%) because of tolerability issues, 2 (10.53%) as a patient/family choice, 2 (10.53%) because of unknown reasons and 4 (21.05%) because of other reasons (medical decision [*n* = 3] and acute psychotic symptoms [*n* = 1]).

An improvement in schizophrenia severity was observed, based on CGI-S reported at the inclusion visit (i.e., following LUR therapy) compared to those reported at the index date (i.e., LUR treatment initiation). In fact, the proportion of study patients defined *markedly ill* and *severely ill* decreased from 17.07% and 29.27% at the index date (start of LUR) to 9.76% and 4.88%, respectively, at the study inclusion visit. Likewise, the percentage of study subjects defined *borderline mentally ill* and *mildly ill* increased (i.e., improved) from 4.88% and 2.44% at the index date to 36,59% and 29.27%, respectively, at the inclusion visit (Tables 2 and 3).

**Table 3:** Clinical Global Impression—Severity scale (CGI-S) at the inclusion visit

	Total (*N* = 41) *n* (%)
CGI-S measurement	
Borderline mentally ill	15 (36.59)
Mildly ill	12 (29.27)
Moderately ill	8 (19.51)
Markedly ill	4 (9.76)
Severely ill	2 (4.88)

Percentages were computed on eligible population

*CGI-S* Clinical Global Impression—Severity scale

### Concomitant medications/treatments

At least one concomitant medication was reported for 40 patients (40, 97.56%). The concomitant medications are reported in Table 4.

**Table 4:** Concomitant medications

	Total (*N* = 41) *n* (%)
Number of patients with any concomitant medication	40 (97.56)
WHO ATC 2nd level/preferred term	
Anti-Parkinson drugs	1 (2.44)
Anticholinergic agents	1 (2.44)
Antiepileptics	4 (9.76)
Antiepileptics	1 (2.44)
Gabapentin	2 (4.88)
Topiramate	2 (4.88)
Antihistamines for systemic use	1 (2.44)
Antihistamines	1 (2.44)
Psychoanaleptics	19 (46.34)
Antidepressants	19 (46.34)
Psycholeptics	37 (90.24)
Anxiolytics	10 (24.39)
Aripiprazole	4 (9.76)
Benzodiazepine derivatives	17 (41.46)
Brexpiprazole	3 (7.32)
Cariprazine	1 (2.44)
Clotiapine	2 (4.88)
Clozapine	7 (17.07)
Haloperidol	4 (9.76)
Olanzapine	7 (17.07)
Paliperidone	1 (2.44)
Pipamperone	1 (2.44)
Psychotropic agents	24 (58.54)
Quetiapine	9 (21.95)
Risperidone	6 (14.63)

Concomitant medications were defined as therapies ending or ongoing after the start of study treatment

Percentages were computed on eligible population

*ATC* Anatomical Therapeutic Class

Ten patients (24.39%) received at least one concomitant non-pharmacological therapy for schizophrenia: 4 patients (9.76%) had group or individual psychoeducation therapy, 3 (7.32%) received rehabilitation/occupational support, 2 (4.88%) had group or individual psychotherapy and 1 (2.44%) received institutional support.

### Adverse events

At least one adverse drug reaction (ADR) possibly related to LUR treatment was reported for 6 patients (14.63%), none of which was serious. One patient (2.44%) underwent an additional diagnostic procedure (Magnetic Resonance Imaging) due to an ADR (hyperprolactinemia). Three patients (7.32%) had an additional visit due to an ADR. One of these 3 patients was the subject who received the Magnetic Resonance Imaging because of hyperprolactinemia, who was referred to an endocrinologist. The other two were patients who received an additional day hospital visit because of extrapyramidal symptoms”.

ADRs possibly related to LUR treatment consisted of akathisia (7.32%), extrapyramidal disorder (4.88%), hyperprolactinemia (2.44%), restlessness (2.44%), and galactorrhea (2.44%).

## Discussion

We aimed to assess treatment persistence rates with LUR in a group of patients with schizophrenia treated in real-world Italian clinical practice and found that 78% were persistent to LUR treatment for at least 6 months and 58% were persistent for at least 18 months. Our persistence rate was relatively high compared to other studies such as the CATIE trial [1], where only about 25% (371 of 1432) of the patients who received at least one dose of study medication (oral olanzapine, perphenazine, quetiapine, risperidone, or ziprasidone) were persistent to their oral treatment for at least 18 months.

In an observational, prospective follow-up of 69 patients consecutively prescribed lurasidone in a large inner-city National Health Service mental health trust in London [3], 45 patients (65%) were known to have discontinued lurasidone at 1 year. Interestingly, most non-persistent patients discontinued LUR because of a perceived inefficacy, which might be related to the unusually high proportion of treatment-resistant subjects in the study sample [6].

In fact, while none of the clinical and demographic variables considered in our study had a statistically significant relationship with LUR treatment persistence, in the study conducted in London, patients who were not treatment-resistant had a substantially reduced risk of discontinuation. Of interest, higher LUR doses were a positive predictive factor for treatment persistence [6].

A study involving 146 Medicaid insured (52.1% male, mean age 43.5 years) and 63 commercially insured (46.1% male, mean age 40.0 years) patients treated with lurasidone evaluated variables such as medication possession ratio (MPR), mean time to discontinuation, and discontinuation rate [7]. In the Medicaid group, patients receiving lurasidone exhibited a significantly lower discontinuation rate compared to those treated with all other antipsychotics (49.3% versus 62.3–68.3%, all *p* < 0.05), the mean time to discontinuation with lurasidone was significantly longer than with ziprasidone (*p* < 0.05), and the MPR was 0.60 for lurasidone, versus 0.41–0.48 for other antipsychotics (all *p* < 0.05). In the commercially insured population, the discontinuation rate (44.4%) was lower for patients on lurasidone compared to patients on all other antipsychotics except risperidone (*p* < 0.05), the mean time to discontinuation was longer for lurasidone compared to other antipsychotics, and the MPR for lurasidone (0.61) was higher than the MPR for quetiapine (0.44) and ziprasidone (0.43) (both *p* < 0.05) [7].

The relatively high persistence rate to LUR in our and other studies may reflect LUR’s relatively favorable balance between efficacy and tolerability. Indeed, LUR has a relatively favorable metabolic side effect profile, and has been recommended as one of the choices when hoping to reduce the risk of metabolic adverse events [8, 9]. For instance, in a double-blind, 12 month, active-controlled study testing the long-term tolerability and safety of lurasidone in schizophrenia [10], a higher proportion of subjects treated with risperidone had an increase in weight of at least 7% compared to LUR (14% for risperidone vs. 7% for lurasidone). Also, the median increase in prolactin was significantly higher for risperidone (*p* < 0.001), while a similar improvement in efficacy measures and similar rates of relapse were observed with both medications. All-cause discontinuation rates were higher for lurasidone versus risperidone but long-term treatment with lurasidone was generally well tolerated.

Interestingly, an improvement in schizophrenia severity, according to the CGI-S, was observed in this study, as highlighted by the reduction in the proportion of patients being defined markedly ill and severely ill, as well as the increased percentage of patients being defined borderline mentally ill and mildly ill, following LUR therapy, compared to before LUR initiation.

In our study, lurasidone was relatively well tolerated, with at least one adverse drug reaction possibly related to LUR being reported for 6 patients (14.63%). ADRs consisted of akathisia (7.32%), extrapyramidal disorder (4.88%), hyperprolactinemia (2.44%), restlessness (2.44%), and galactorrhea (2.44%). None of the events were serious.

The main limitations of this study include its observational retrospective nature, which may bring the risk of patient selection bias, incomplete or missing data, lack of internal validity (no control group), difficulty in interpreting or verifying documented information, and variability among patients in the quality of documentation. Also, the information collected in the eCRF was limited to what was available in the medical records at the participating centers and did not include data related to health services received outside of that setting.

Additionally, the study was conducted in 5 Italian sites and the generalizability of our findings in other healthcare systems is difficult to ascertain. Moreover, the study sample size was relatively small, which may have prevented the observation of the impact on persistence by variables (e.g., rare side effects) that are infrequent. Finally, the lack of significant correlations between persistence and baseline demographic or clinical variables may have been simply due to the lack of statistical power, given the small sample size, and the fact that most patients were receiving concomitant medications may have had an impact on treatment persistence.

Nonetheless, a real life, non-interventional retrospective design allows the possibility to study conditions that are less likely to be influenced by the constraints of a more structured study design and that are often more representative of the patients and clinical scenarios that are encountered in the real-world practice.

## Conclusions

The persistence rate to LUR of patients with schizophrenia treated in the real-world setting of 5 Italian centers was relatively high with 78% and 58% of patients that were still on LUR after 6 and 18 months of treatment, respectively. This high persistence may reflect LUR’s relatively favorable balance between efficacy and tolerability as well as a generally favorable patients’ satisfaction and acceptance. Larger studies are needed to confirm our findings and further research is required to identify factors that can predict the likelihood of treatment continuation with lurasidone.

## Data Availability

The datasets used and/or analyzed during the current study are available from the corresponding author on reasonable request.

## Abbreviations

ADR: Adverse drug reaction
CATIE: Clinical antipsychotic trials of intervention effectiveness
CGI-S: Clinical Global Impression—Severity scale
LUR: Lurasidone
MPR: Medication possession ratio
